# Hematological and Serum Biochemical Values of Free-Ranging Roe Deer (*Capreolus capreolus*) in Poland

**DOI:** 10.3390/ani13020242

**Published:** 2023-01-09

**Authors:** Mirosław Karpiński, Piotr Czyżowski, Sławomir Beeger, Marian Flis

**Affiliations:** Department of Animal Ethology and Wildlife Management, University of Life Sciences in Lublin, Akademicka 13, 20-950 Lublin, Poland

**Keywords:** *Capreolus capreolus*, blood hematology, serum biochemistry indicators, capture

## Abstract

**Simple Summary:**

In wildlife management, it is often necessary to capture live animals for resettlement or to collect biological samples. The method of trapping deer significantly affects the values of the vital parameters determining their welfare. This study provides hematological and serum biochemical intervals for physically captured free-ranging roe deer. The RBC, HCT, and Hb values were significantly lower in males than in females. These results may be helpful to create reference ranges for roe deer.

**Abstract:**

This study presents the hematological and serum biochemical values of physically captured roe deer (*Capreolus capreolus*). The study material was blood sampled from roe deer captured with the use of a specialist trap mesh and no anesthesia. Blood samples were collected from 122 roe deer from January to April in 2016, 2017, and 2018 in the Lublin region of Poland. The hematological and biochemical variables were determined with results showing statistically significant differences between the mean values of RBC, HCT, and HB according to sex. Reference intervals should be more specific to the broad physical versus chemical capture categories.

## 1. Introduction

Currently, the roe deer (*Capreolus capreolus*) is one of the most numerous species in the Cervidae family in Europe [1,2,3]. The species is found in various climatic zones throughout the European continent, except for Ireland, some Mediterranean islands, and northern Russia [4,5]. At present, roe deer density is increasing throughout Europe due to climate change and changes in agricultural ecosystems [6,7,8]. The analysis of roe deer population dynamics indicates that the increase in the population size of the species will persist [2,9,10]. This upward trend is associated with ecological and economic implications, e.g., higher compensations for hunting-related damage [11,12,13] and increasing numbers of road (or even airfield) collisions with roe deer [14,15]. This necessitates the rational and precise management of roe deer populations, taking into account the latest knowledge in veterinary medicine, ecology, and biology. Knowledge of reference hematological and serum biochemical values are becoming more intrinsic to decisions on the fate of captured live deer. Hematology and serum biochemistry data are useful indicators of the animal’s health status and can be used as early warning signs of possible problems in the population associated with winter feeding and parasitic infestations [16]. There are relatively sparse reports on the hematological and biochemical indices of roe deer blood. The variables are most often determined after pharmacological immobilization of the animal or in small samples [17,18,19]. Such results may be influenced by the trapping method, season, analytical method, age, and sex [20].

The aim of this study is to report the hematological and serum biochemical values for physically captured roe deer (*Capreolus capreolus*).

## 2. Materials and Methods

### 2.1. Study Area

The roe deer were captured in the Lublin region of Southeast Poland. The furthest points of the area are as follows: north 52°24′37.46″ N, south 50°14′39.58″ S, west 21°23′58.11″ W, and east 24°08′45.25″ E. The study area extends over a distance of 2°09′57.88″ from south to north and 2°44′47.14″ from west to east. The study area varies in terms of climate, which is classified as moderate transitional in the northern part and submontane in the southern plains and valleys. The annual amount of precipitation depending on the elevation above sea level ranges from 500 to 600 mm in the north and up to 700–800 mm in the south. The average annual temperature for most of the area is about 7.5 °C. The growing period lasts from 208 to 220 days. Forests cover 23% of the area and are characterized by great diversity in terms of size and species composition.

### 2.2. Sample Collection

Blood used for the determinations was sampled from roe deer captured with the use of a specialized trap mesh but with no use of pharmacological agents. The roe deer were herded and then a net was cast over them using an air launcher. It took an average of 10–15 min to immobilize the animals. The captured roe deer were removed from the trap and physically restrained in lateral recumbency on the ground during handling by qualified personnel. A veterinarian performed a clinical examination and collected blood from the jugular vein. The handling consisted of identification of the sex and health assessment through nutritional status, hair coat condition, and absence of external injuries. A total of 122 samples from 74 males and 48 females were suitable for analysis. Blood was sampled from January to April in 2016, 2017, and 2018. All procedures were carried out during vet interventions, including catching roe deer in municipal parks (three samples) and glider airports (one sample). No ethical permit for animal experiments had to be obtained. All applicable international and/or institutional guidelines for the care and use of animals were followed. The blood samples were collected as part of the routine surveillance program performed by vets ordered by an administrative unit (City Council). According to the act of 15 January 2015 on the protection of animals used for scientific or educational purposes in Poland, the approval of an Ethics Committee is not required to perform activities on animals obtained for purposes other than research (e.g., routine trapping and relocation procedures, post-slaughter studies if the slaughter was not the purpose of the study).

Blood was collected in a serum tube (10 mL syringes and 0.8 mm needles) with a clot activator for serum biochemistry and an ethylenediamine tetra-acetic acid (EDTA) tube for hematology. Blood samples with the clot activator were centrifuged (1500× *g* at 4 °C for 10 min) within 2 h of collection, and the serum was stored at −18 °C prior to biochemistry analysis. Samples for hematology were refrigerated at +4 °C and analyzed within 12 h. The hematological variables were analyzed on an Orphee Mythic-18 automated hematology analyzer using domestic ruminants, i.e., sheep and goat settings [21], for the following hematologic variables: white blood cell count (WBC), red blood cell count (RBC), hematocrit (HCT), hemoglobin (Hb), platelet count (PLT), lymphocytes (Lym), monocytes (Mon), and granulocytes (Gra). Sera were analyzed using an ACCENT-200 PZ Cormay automatic biochemistry analyzer and original reagents from the manufacturer for the serum activities of alanine aminotransferase (ALT), aspartate aminotransferase (AST), alkaline phosphatase (AP), and amylase (AMY), and serum concentrations of the total protein (tPROT), urea concentration (Urea), creatinine (Crea), cholesterol (Chol), and total bilirubin (tBIL). The analyses were performed at the Central Laboratory of the Center of Small Animals in Lublin.

### 2.3. Statistical Analysis

The normality of the distribution of the analyzed traits was tested using the Shapiro–Wilk test. In order to eliminate outliers, Dixon’s range statistic was used [22]. Statistical significance was set at *p* ≤ 0.05. The significance of differences between sexes was assessed with the *t*-test for normally distributed variables and the non-parametric Mann–Whitney U rank test for non-normally distributed variables. All the analyses were performed using Statistica 13.3 (TIBCO Software Inc., Palo Alto, CA, USA).

## 3. Results

The majority of the analyzed hematological variables were normally distributed, with the exception of hemoglobin, the platelet count, and granulocytes. In the case of biochemical variables, only cholesterol and total protein had a normal distribution. The RBC, HCT, and Hb values were significantly lower in males than in females (Table 1).

## 4. Discussion

The analysis of hematological variables is an important tool for monitoring the health status of wild animals. However, there is scarce information available on the biochemical profiles of roe deer. The data obtained in the present study are difficult to compare with results from other studies of deer due to the individual variability in enzyme activity and differences in the trapping methods, the season of the year, and the age of the roe deer. There are objective difficulties in the assessment of results obtained with the use of different techniques and expressed in different units [23]. As reported in the literature [22], establishing the health status of wild-caught species is especially challenging, and protocols should be rigorously established to minimize variations caused by including subjects of indeterminate health.

This study provides reference values for the hematological and serum biochemical variables for physically captured roe deer, with separate intervals for males and females for RBC, HCT, Hb, and Urea. The capture method and resulting stress significantly affect the values of these variables in roe deer [18], leading to the recommendation of establishing different reference ranges for each capture method [17,24].

The characteristics of the roe deer analyzed in the present study did not differ from those reported by other authors in studies conducted on deer [25,26,27,28], though there were slight differences in the increase in some blood count variables, e.g., the red blood cell count (RBC) and hematocrit (PCV, HCT). The increase in these values may result from splenic contraction associated with an increase in the plasma catecholamine concentration [29,30] which is a consequence of the acute stress reaction experienced by the animal being trapped for blood collection. Moreover, as reported by Küker et al. [17], after Chapple et al. [31] and Weiss and Wardrop [32], different hemoglobin concentrations and hematocrit values in females may be associated with the activity of female sex hormones.

Our results related to the renal profile variables (serum urea and creatinine concentrations) were similar to the concentration reported by Küker et al. [17] and higher than those shown by Severin et al. (2012). As suggested by Marco and Lavin [25], this may be related to the secondary stress response to capture and restraint [33]. The liver profile indices (serum cholesterol and bilirubin concentration) were similar to those reported by Küker et al. [17], although our results obtained in the physically captured roe deer were closer to the findings shown by these authors in the examination of chemically immobilized individuals. The serum ALT activity was within the range specified by Küker et al. [17] for physically captured deer, while the serum AST activity was substantially higher than that reported by these authors, which may indicate the stress experienced by the roe deer in the present study.

## 5. Conclusions

This study provides values for the hematological and serum biochemical variables of roe deer in the absence of clinical symptoms. Separate reference intervals should be provided for different capture methods, especially in such a stress-sensitive species as roe deer. The study showed statistically significant gender differences in such indicators as RBC, HCT, and Hb. Separate reference intervals should be provided for different capture methods (physical and chemical) but also should take into account the differences which may occur depending on the physical capture method (i.e., stress and its effects are different for a drive-net captured roe deer and a box-trap captured roe deer). These results may be helpful to create reference ranges for roe deer.

## Figures and Tables

**Table 1:** Average values (the mean ± standard error (n)) of the morphological and biochemical indicators of roe deer blood.

Value	Total Mean	95% Confidence Interval	*p*-Value (Type of Significance Test between Sex)	Female	Male
RBC (10^12^/L)	13.6 ± 0.7 (53)	12.2–15,0	0.0074 (t)	16.5 ± 1.2 (15)	12.4 ± 0.8 (38)
HCT (L/L)	0.56 ± 0.03 (54)	50.8–61.2	0.0163 (t)	0.66 ± 0.05 (15)	0.52 ± 0.03 (39)
Hb (g/L)	158 ± 5.4 (54)	14.7–16.9	0.0310 (U)	178 ± 8.0 (15)	150 ± 6.4 (39)
PLT (10^9^/L)	221 ± 19.1 (52)	182–259	0.1431 (U)		
WBC (10^9^/L)	29.0 ± 1.7 (54)	25.6–32.4	0.0891 (t)		
Lym (10^9^/L)	20.7 ± 1.6 (30)	54.4–69.4	0.4531 (t)		
Mon (10^9^/L)	2.2 ± 0.3 (29)	5.1–7.5	0.0780 (t)		
Gra (10^9^/L)	9.9 ± 1.4 (30)	22.9–36.2	0.1817 (U)		
ALT (U/L)	72.0 ± 9.1 (121)	53.6–89.5	0.1466 (U)		
AST (U/L)	496 ± 54 (121)	386–600	0.1587 (U)		
Urea (mmol/L)	11.2 ± 0.9 (121)	26.9–36.8	0. 1150 (U)		
Crea (μmol/L)	148 ± 3.6 (122)	1.6–1.8	0.5716 (U)		
AP (U/L)	48.7 ± 4.1 (59)	40.6–56.9	0.4842 (U)		
AMY (U/L)	43.7 ± 4.5 (48)	34.6–52.8	0.4903 (U)		
Chol (μmol/L)	1.33 ± 0.04 (117)	48.5–54.5	0.3587 (t)		
tPROT (g/L)	58.8 ± 1.5 (59)	5.6–6.2	0.3273 (t)		
tBIL (μmol/L)	18.8 ± 10.9 (48)	0.2–2.4	0.9550 (U)		

*p*-value for the test of significance of the differences between the mean values for the sexes. Significant differences at *p* ≤ 0.05 are shown in bold font, (t) = *t*-test, (U) = Mann–Whitney U test, white blood cell count (WBC), red blood cell count (RBC), hematocrit (HCT), hemoglobin (Hb), platelet count (PLT), lymphocytes (Lym), monocytes (Mon), granulocytes (Gra), alanine aminotransferase (ALT), aspartate aminotransferase (AST), alkaline phosphatase (AP), amylase (AMY), total protein (tPROT), urea concentration (Urea), creatinine (Crea), cholesterol (Chol), and total bilirubin (tBIL).

## Data Availability

The datasets generated during and/or analyzed during the current study are available from the corresponding author upon reasonable request.
